# Complex Associations Between Systolic Left Atrial and Left Ventricular Deformations in Healthy Adults—Detailed Analysis from the Three-Dimensional Speckle-Tracking Echocardiographic MAGYAR-Healthy Study

**DOI:** 10.3390/life15020287

**Published:** 2025-02-12

**Authors:** Attila Nemes, Barbara Bordács, Nóra Ambrus, Csaba Lengyel

**Affiliations:** Department of Medicine, Albert Szent-Györgyi Medical School, University of Szeged, H-6725 Szeged, Hungary; bordacs.barbara.aniko@med.u-szeged.hu (B.B.); ambrusnora@gmail.com (N.A.); lecs@in1st.szote.u-szeged.hu (C.L.)

**Keywords:** left atrial, left ventricular, volume, strain, three-dimensional, speckle-tracking echocardiography, healthy

## Abstract

**Introduction.** Volumetric changes in the left atrium (LA) and left ventricle (LV) are strongly associated in healthy circumstances, as recent three-dimensional speckle-tracking echocardiographic (3DSTE) studies confirmed. However, the complex relationship of LA and LV deformation in systole has never been assessed in healthy individuals. The present study purposed to perform comparative simultaneous analysis of systolic LA and LV strains in healthy adults by 3DSTE. **Methods.** The study consisted of 165 healthy adults (mean age: 33.2 ± 12.3 years; 90 males). Complete two-dimensional Doppler echocardiography with 3DSTE was performed in all cases. **Results.** The increase in global LA radial strain (RS) and longitudinal strain (LS) showed no associations with LV strains. The largest global LA circumferential strain (CS) was associated with the largest basal LV-RS. Lowest basal and global LV-CS could be demonstrated in the presence of mean global LA-CS as compared to the presence of lower than mean global LA-CS. Global LA-RS showed an increase, with global LV-RS being largest when global LV-RS was the largest. Basal and global LA-RS were largest in the case of lowest global LV-CS. Basal LA-LS was largest in the case of mean global LV-CS. With the increase in global LV-LS, basal and global LA-LS showed an increase but only up to a point; in the case of larger than mean global LV-LS, no further increase was detected in basal and global LA-LS. **Conclusions**. Complex associations between simultaneously assessed LA and LV deformations represented by 3DSTE-derived strains could be demonstrated in healthy adults.

## 1. Introduction

The new cardiovascular imaging procedures enable a detailed analysis of cardiac mechanics that was not possible with previous techniques. The strong development in echocardiography in recent decades has meant that, in addition to the previously available methods, wall contractility can now be examined and featured in detail in addition to changes in the volumes of the heart chambers respecting the cardiac cycle. Three-dimensional (3D) speckle-tracking echocardiography (STE) is one of the most advanced methods, which, with the help of virtual 3D models of ventricles and atria, enables the simultaneous volumetric and strain analysis of any cardiac chamber, even allowing physiologic tests to be performed on healthy subjects [1,2,3,4,5]. Volumetric changes in the left atrium (LA) and left ventricle (LV) are strongly associated in healthy circumstances, as a recent 3DSTE study confirmed [6]. However, the complex relationship of LA and LV deformation in systole has never been assessed in healthy individuals by 3DSTE. Therefore, the present study aimed to perform a comparative simultaneous analysis of systolic LA and LV strains in healthy adults by 3DSTE.

## 2. Subjects and Methods

### 2.1. Subjects

The study consisted of 165 healthy adult subjects (mean age: 33.2 ± 12.3 years; 90 males). Subjects were considered to be healthy if acute or chronic disorders, pathological conditions, drug usage, smoking, obesity, and any other condition that could affect findings could be excluded. All routine echocardiographic variables were normal and were within the normal reference ranges. None of them were athletes or performed yoga 1 week before the echocardiographic study. Electrocardiographic (ECG) and laboratory results were normal. M-mode, two-dimensional (2D) and Doppler echocardiography, and 3DSTE were obtained in all subjects. This retrospective cohort study serves as a part of the ‘Motion Analysis of the heart and Great vessels bY three-dimension Al speckle-tRacking echocardiography in Healthy subjects’ (MAGYAR-Healthy) Study, which was organized at the University of Szeged partly for the physiologic analysis of the relationship of 3DSTE-derived parameters in healthy circumstances (in the Hungarian language, ‘Magyar’ means ‘Hungarian’). The study was conducted in accordance with the Declaration of Helsinki (as revised in 2013). The Institutional and Regional Human Biomedical Research Committee of University of Szeged, Hungary (No.: 71/2011) approved the study, and informed consent was provided by all healthy individuals.

### 2.2. Two-Dimensional Doppler Echocardiography

In healthy cases, a full two-dimensional (2D) echocardiographic examination was carried out, for which a Toshiba Artida^TM^ (Toshiba Medical Systems, Tokyo, Japan) cardiac ultrasound device attached to a PST-30BT (1–5 MHz) phased-array transducer was used. Following LA and LV quantifications, early (E) and late (A) diastolic mitral inflow velocities and their ratio (E/A) were determined by Doppler. Moreover, larger than grade 1 regurgitations and significant stenoses on any valves were excluded by Doppler-based assessments [7]. 

### 2.3. Three-Dimensional Speckle-Tracking Echocardiography

3DSTE was performed in all cases immediately after the 2D Doppler echocardiography in 2 stages [1,2,3,4,5]. First, data were acquired using the same device by exchanging the transducer to a PST-25SX matrix phased-array transducer; in cases being in sinus rhythm, subjects were lying in a left lateral decubitus position. Following image optimizations (gain, magnitude, etc.), 3D echocardiographic datasets were acquired from the apical window within a single breath-hold. Also, for the best image quality, 6 subvolumes were acquired within 6 heart cycles, which were merged together automatically for a 3D full volume dataset. As a second step, data were analyzed offline by a vendor-provided 3D Wall Motion Tracking software (version 2.7, Ultra Extend, Toshiba Medical Systems, Tokyo, Japan) [1,2,3,4,5]. 

### 2.4. DSTE-Derived LA/LV Volumes and Strains

**Figure 1:**
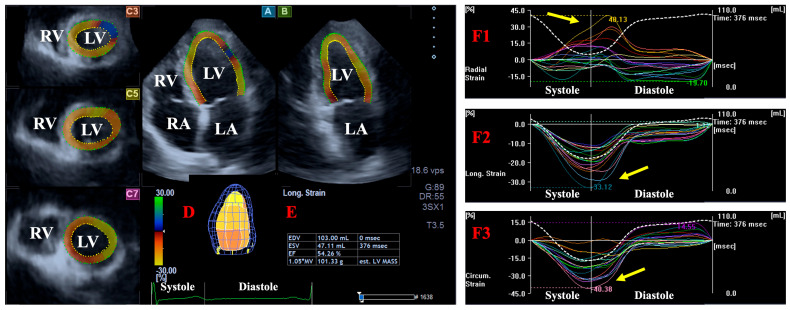
Three-dimensional (3D) speckle-tracking echocardiography-derived determination of left ventricular (LV) strains: apical four-chamber (A) and two-chamber (B) long-axis views and short-axis views at apical (C3), mid-ventricular (C5), and basal LV levels (C7) are demonstrated together with a virtual 3D model of LV (D) and calculated LV volumetric data (E). Global (white line) and segmental (colored lines) time–radial (F1), –longitudinal (F2), and –circumferential (F3) LV strain curves are presented together with time–LV volume change curves (dashed white line). Yellow arrow represents maximum LV strains in end-systole. Abbreviations. EDV, end-diastolic volume; EF, ejection fraction; ESV, end-systolic volume; RA, right atrium; RV, right ventricle; LA, left atrium; LV, left ventricle.

**Figure 2:**
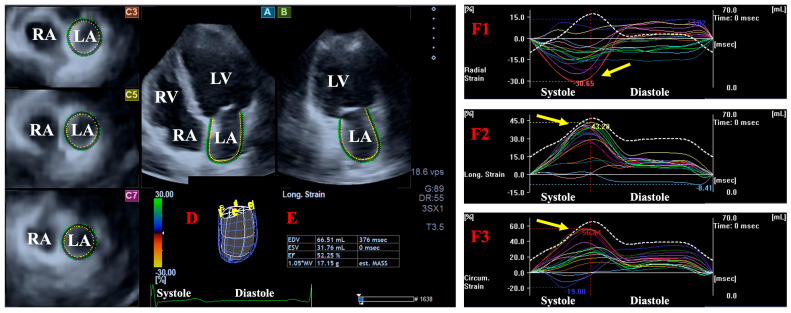
Three-dimensional (3D) speckle-tracking echocardiography-derived determination of left atrial (LA) strains: apical four-chamber (A) and two-chamber (B) long-axis views and short-axis views at basal (C3), mid-atrial (C5), and superior LA levels (C7) are demonstrated together with a virtual 3D model of LA (D) and calculated LA volumetric data (E). Global (white line) and segmental (colored lines) time–radial (F1), –longitudinal (F2), and –circumferential (F3) LA strain curves are presented together with time–LA volume change curves (dashed while line). Yellow arrow represents end-systolic peak LA strains. Abbreviations. EDV, end-diastolic volume; EF, ejection fraction; ESV, end-systolic volume; RA, right atrium; RV, right ventricle; LA, left atrium; LV, left ventricle.

Using the acquired 3D datasets, apical 2-chamber (AP2CH) and 4-chamber (AP4CH) long-axis views and 3 short-axis views in basal, mid-atrial, and superior regions for LA and apical, midventricular, and basal regions for LV were created following image plane optimizations. In the case of LV, endocardial lateral and septal edges of the MA and LV apex were defined by setting reference points and then following a sequential analysis, and a virtual 3D model of LV was created. Similarly, multiple reference points were identified around LA from edge to edge of MA, and a sequential analysis was carried out. The pulmonary veins and the LA appendage were excluded from measurements. For LA, the following volumes were measured respecting the cardiac cycle [8] (Figure 1 and Figure 2):

- V_max_—end-systolic maximum LA volume, measured just before mitral valve opening (largest LA volume).
- V_preA_—LA volume before atrial contraction in early diastole, at the time of the P wave on the ECG.
- V_min_—late diastolic minimum LA volume, measured just before mitral valve closure (smallest LA volume).

For LV, the following parameters were measured respecting the heart cycle [9]:

- LV volume in end-diastole (EDV).
- LV volume in end-systole (ESV).
- Ejection fraction (EF) of the LV.

For both LA and LV, several strains, quantitative features of wall contractility, were measured in end-systole using 3D echocardiographic datasets and casts of LA and LV. On 3DSTE-derived LA strain curves, the first peak represented end-systolic LA reservoir function. The following global (featuring the whole chamber) LA/LV strains were measured and basal regional LA/LV strains were calculated from 6 basal segmental LA/LV strains [10,11] (Figure 1 and Figure 2):

- LA/LV radial strain (LA/LV-RS), featuring the thinning/thickening of the myocardial tissue.
- LA/LV circumferential strain (LA/LV-CS), featuring the widening/narrowing of the myocardial tissue.
- LA/LV longitudinal strain (LA/LV-LS), featuring the lengthening/shortening of the myocardial tissue.

### 2.5. Statistical Analysis

Continuous and categorical variables are shown as mean ± standard deviation (SD) and *n* (%) formats, respectively. Statistical significance was considered to be present when *p* was less than 0.05. Analysis of variance (ANOVA), an independent sample *t*-test, and Chi-squared tests were used for comparisons, where appropriate. Intra- and inter-observer agreements were tested by interclass correlation coefficients (ICCs). Version 22^nd^ for SPSS software (SPSS Inc., Chicago, IL, USA) was used for statistical analyses.

## 3. Results

### 3.1. Two-Dimensional Doppler Echocardiography

The LA diameter measured in parasternal long-axis view (35.9 ± 3.8 mm), LV end-diastolic diameter (48.3 ± 3.1 mm) and volume (105.9 ± 21.3 mL), LV end-systolic diameter (32.0 ± 3.3 mm) and volume (35.9 ± 8.9 mL), interventricular septum (9.1 ± 1.6 mm) and LV posterior wall (9.2 ± 1.3 mm), and LV ejection fraction (65.5 ± 4.3%) were in the normal range. The mean E/A proved to be 1.34 ± 0.32. Valvular regurgitation ≥ grade 1 or stenosis on any valves were not present in any healthy adults. 

### 3.2. Subject Classifications

The average ± SD values of 3DSTE-derived LA and LV strains of healthy individuals are depicted in Table 1. Healthy subjects were classified into three groups according to the normal global LA-RS, LA-CS, LA-LS, LV-RS, LV-CS, and LV-LS: estimated average − SD served as the lower (−6.6%, 18.2%, 17.2%, 16.1%, −22.4%, and −13.8%, respectively) and estimated average + SD served as the higher −23%, 48.8%, 35%, 34.9%, −32.6%, and −18.6%, respectively) cut-off values (Table 1). 

### 3.3. 3DSTE-Derived LA Volumes and Strains in LA Strain Subgroups

Increased global LA-RS was associated with increased systolic LA-V_max_ up to a point with unchanged diastolic LA-V_preA_ and LA-V_min_. Increased global LA-CS and LA-LS were associated with unchanged LA-V_max_ and reduced LA-V_preA_ and LA-V_min_. Increased global LA strains were associated with increase of other global (not all basal) LA strains (Table 2).

### 3.4. 3DSTE-Derived LV Volumes and Strains in LA Strain Subgroups

Global LA-RS and LA-LS showed no relationship with LV volumes. An increase in LV-EDV and LV-ESV was seen in the presence of mean global LA-CS as compared to the presence of lower than mean global LA-CS, which showed no further increase if global LA-CS was larger than mean. The lowest LV-EF was present in the presence of mean global LA-CS. LV mass did not change with global LA-CS and LA-LS, but was largest in case of largest global LA-RS. The increase in global LA-RS and LA-LS showed no associations with LV strains. The largest global LA-CS was associated with the largest basal LV-RS. The lowest basal and global LV-CS could be demonstrated in the presence of mean global LA-CS as compared to the presence of lower than mean global LA-CS (Table 2).

### 3.5. 3DSTE-Derived LA Volumes and Strains in LV Strain Subgroups

Global LV-RS and LV-LS showed no relationship with LA volumes. The lowest LA-V_max_ was seen in the presence of the highest global LV-CS. Global LA-RS showed an increase, with global LA-RS being the largest when global LV-RS was the largest. Basal and global LA-RS were the largest in the case of the lowest global LV-CS. Basal LA-LS was the largest in the case of mean global LV-CS. With an increase in global LV-LS, basal and global LA-LS showed an increase but only up to a point; in the case of larger than mean global LV-LS, no further increase in basal and global LA-LS was detected (Table 3).

### 3.6. 3DSTE-Derived LV Volumes and Strains in LV Strain Subgroups

LV-EF was increased—to a different extent—in the case of larger global LV-RS due to non-significantly increased LV-EDV, in the case of larger global LV-CS due to reduced LV-ESV, and in the case of larger global LV-LS due to reduced LV-EDV and LV-ESV. LV mass did not change with global LV-RS and LV-CS, but was largest in case of lowest global LV-LS. Increased global LV-RS and LV-CS were associated with increase of other basal and global LV strains, except basal and global LV-LS. The largest global LV-LS was associated with increased global LV-RS and LV-CS (Table 3).

### 3.7. Correlations

Significant correlations could be demonstrated between global LA-RS and global LA-CS (r = −0.29; *p* < 0.001), global LA-RS and global LA-LS (r = −0.23; *p* < 0.05), global LA-CS and global LA-LS (r = 0.45; *p* < 0.001), global LV-RS and global LV-CS (r = 0.53; *p* < 0.001), global LV-RS and global LV-LS (r = −0.30; *p* < 0.001), and global LV-CS and global LV-LS (r = 0.18; *p* = 0.02). 

There were no significant correlations between global LA-RS and global LV-RS (r = −0.13; *p* =0.07), global LA-RS and global LV-CS (r = −0.11; *p* = 0.17), global LA-RS and global LV-LS (r = −0.08; *p* = 0.32), global LA-CS and global LV-RS (r = 0.01; *p* = 0.89), global LA-CS and global LV-CS (r = −0.06; *p* = 0.71), global LA-LS and global LV-RS (r = 0.01; *p* = 0.86), global LA-LS and global LV-CS (r = −0.09; *p* = 0.23), and global LA-LS and global LV-LS (r = −0.11; *p* = 0.22).

### 3.8. Reproducibility of 3DSTE-Derived LA/LV Parameters

Intra-observer ICCs for Vmax, VpreA, Vmin, global LA-RS, LA-CS, LA-LS, LV-EDV, LV-ESV, global LV-RS, LV-CS, and LV-LS were 0.97, 0.85, 0.95, 0.76, 0.76, 0.57, 0.92, 0.92, 0.85, 0.83, and 0.81, respectively (*p* < 0.05 for all values). Inter-observer ICCs for the same parameters proved to be 0.96, 0.85, 0.96, 0.67, 0.76, 0.66, 0.91, 0.93, 0.82, 0.77, and 0.77, respectively (*p* < 0.05 for all values).

## 4. Discussion

The engine of central circulation is an organic unit of LA and LV [12]. During the cardiac cycle, both LA and LV have special volumetric and functional characteristics. LA is a reservoir in systole, when its volume is the largest (LA-V_max_), with wall thinning in the radial direction (LA-RS with a minus sign), widening in the circumferential, and lengthening in the longitudinal directions (LA-CS and LA-LS with plus signs), as seen from the 3DSTE analysis [13,14,15,16]. At the same time, LV has the smallest volume at end-systole (LV-ESV) with opposite LV contractility features to LA: wall thickening in the radial direction (LV-RS with a plus sign), narrowing in the circumferential, and shortening in the longitudinal directions (LV-CS and LV-LS with minus signs). In early diastole, LA is a conduit allowing blood flow from the pulmonary veins to LV via the mitral valve with LA-V_preA_, while in late diastole, it is a booster pump with an active contraction and the lowest LA volume (LA-V_min_). At the same time, LV is filling from LA via the mitral valve, with the largest volume at end-diastole (LV-EDV) [17,18,19]. 

3DSTE is a new cardiovascular imaging technique with the ability of simultaneously evaluating LA and LV strains and volumes using virtually created casts of these heart chambers. It is non-invasive, easy-to-implement, and easy-to-learn, as it is validated for both LA and LV [20,21,22,23,24,25]. Moreover, normal reference ranges and gender- and age-dependency of 3DSTE-derived LA/LV volumes and strains have already been published in the frame of the MAGYAR-Healthy Study [8,9,10,11]. The complex associations between LV volumes (and consequential LV-EF) and LV strains have already been demonstrated in healthy adults [26], just like similar relationships between LA volumes and LA strains [27] LV volumes and LA volumes [6] and strains [28] and LA volumes and LV strains [29]. 

The present study extended this knowledge by evaluating relationships between end-systolic LA and LV deformations represented by unidimensional/unidirectional strains in healthy circumstances as assessed by 3DSTE simultaneously. From LA strains, only LA-GCS showed associations with basal LV-RS and basal and global LV-CS. Of the LV strains, LV-GRS was associated with global LA-RS, and LV-GCS with basal and global LA-RS and basal LA-LS, and associations were found between LV-GLS and basal and global LA-LS. However, LA and LV strains did not correlate directly with each other. 

Based on the literature, it can be said that the LA and LV strains seem to be suitable for assessing the success of certain drug treatments, e.g., angiotensin-converting enzyme inhibitors [30], angiotensin receptor neprilysin inhibitors [31], and beta-blockers [32]. One may wonder how these deformation parameters could serve as biomarkers in the follow-up. Both global LA-LS and LV-LS were found to have significant prognostic power in certain pathologies and healthy adults too [33,34,35,36]. However, their combined values and their complex relationship in these scenarios have never been examined in detail as was done in this study.

Atrial fibrillation (AF) is not just a question of atrial enlargement; it is related to the structural, electrical, and biochemical remodeling of the LA. 2DSTE-derived LA strains allow for the detailed assessment of all phases of LA function in a non-invasive way with a significant prognostic value and determined normal references. Reduced LA strain could be found in patients with AF and associated structural remodeling, fibrosis, and reduced contractility/relaxation [37]. With 3DSTE, the most important limitation is its inability to determine any LA strain; in cases with AF, subjects have to be in sinus rhythm [1,2,3,4,5].

Another interesting area where the significance of the atrio–ventricular interaction presented above may be of interest is the question of heart failure and its extent. The changes in interplay between LA and LV during the heart cycle (atrio–ventricular coupling) may have effects on global heart performance and heart failure [38]. The presented association between LA and LV volumes/strains and the complexity of their changes could theoretically have diagnostic and/or prognostic importance, which requires further investigations.

This study has several implications that are worth considering. First of all, the most important thing is that it would be a mistake to examine the strain parameters characterizing LV/LA wall contractility independently of the volumes. Knowing its physiological background (see the Frank–Starling mechanism) is of essential importance [13,14,15,18,39]. On the other hand, findings from the present study also highlight that the contractility of the myocardial walls of a heart chamber is three-dimensional; accordingly, their extent can be different in the three directions of space, and they can affect the contractility of other heart chambers [2,3,4,18]. Thirdly, although this was not examined in the present study, the situation may be further complicated by the rotational mechanics of LV or valvular sizes (like mitral annular dimensions), as confirmed by previous results from the MAGYAR-Healthy Study [13,17,40]. These facts can draw attention to the importance of clinico-physiological tests similar to the above, and to the analysis of correlations that have not yet been thoroughly investigated under healthy conditions. 

### Limitations

- Although all subjects were considered to be healthy, it could not be excluded with 100% certainty that some individuals had subclinical abnormalities. Follow-up studies later could help in identifying such cases [33,34].
- Only a limited number of healthy cases were involved. A larger number of healthy individuals would have made the whole study more convincing.
- There are differences in quality between 2D echocardiography-derived images and those acquired by 3DSTE. With 2D echocardiography, significantly better image quality can still be achieved due to superior spatial and temporal resolutions, which limits 3DSTE in the clinical practice [1,2,3,4,5]. The 3DSTE-related frame rate is low (31 ± 2 fps), which could affect image quality and results. Moreover, the size of transducer for 3DSTE is significantly larger than that for 2D echocardiography, which could limit data acquisitions. Moreover, for optimal images, six subvolumes during six cardiac cycles were acquired, which increased the chances that stitching and/or motion artifacts would develop and limit image quality.
- According to previous findings, 51% feasibility could be detected in the simultaneous assessment of LA and LV features in the frame of the MAGYAR-Healthy Study, which could also limit the findings [13].
- It was not purposed to validate LA/LV strains as assessed by 3DSTE due to their validated nature [20,21,22,23,24,25].
- Only LA and LV volumes and strains were measured in the present study. Although 3DSTE is able to assess other parameters like rotational features for LV or volumes/strains for the right atrium and ventricle, this study did not purpose such assessments.
- There could be other parameters affecting LA and LV deformations, which were not considered in this study.

## 5. Conclusions

Complex associations between simultaneously assessed LA and LV deformations represented by 3DSTE-derived strains could be demonstrated in healthy adults. 

## Figures and Tables

**Table 1:** Three-dimensional speckle-tracking echocardiography-derived left atrial and left ventricular volumes and end-systolic strains.

Parameters	Measures
**Left atrial volumes and strains**	
**maximum left atrial volume (LA-V_max,_ mL)**	40.9 ± 13.1
**pre-atrial contraction left atrial volume (LA-V_preA,_ mL)**	27.7 ± 11.8
**minimum left atrial volume (LA-V_min,_ mL)**	19.4 ± 8.1
**left atrial global radial strain (LA-GRS, %)**	−14.8 ± 8.2
**left atrial global circumferential strain (LA-GCS, %)**	33.5 ± 15.3
**left atrial global longitudinal strain (LA-GLS, %)**	26.1 ± 8.9
**Left ventricular volumes and strains**	
**left ventricular end-diastolic volume (LV-EDV, mL)**	86.1 ± 22.8
**left ventricular end-systolic volume (LV-ESV, mL)**	36.3 ± 10.4
**left ventricular ejection fraction (LV-EF, %)**	58.1 ± 5.6
**left ventricular mass (g)**	159.2 ± 31.9
**left ventricular global radial strain (LV-GRS, %)**	25.5 ± 9.4
**left ventricular global circumferential strain (LV-GCS, %)**	−27.5 ± 5.1
**left ventricular global longitudinal strain (LV-GLS, %)**	−16.2 ± 2.4

**Table 2:** Left atrial and left ventricular volumes and end-systolic strains in different left atrial strain groups.

	LA-GRS < −6.6% (*n* = 24)	−6.6% ≤ LA-GRS ≤ −23% (*n* = 123)	−23% < LA-GRS (*n* = 18)	LA-GCS < 18.2% (*n* = 25)	18.2% ≤ LA-GCS ≤ 48.8% (*n* = 111)	48.8% < LA-GCS (*n* = 29)	LA-GLS < 17.2% (*n* = 24)	17.2% ≤ LA-GLS ≤ 35% (*n* = 116)	35% < LA-GCS (*n* = 25)
**LA-V_max_ (mL)**	35.8 ± 13.4	41.7 ± 13.2 ∗	42.2 ± 11.4	38.3 ± 11.4	41.9 ± 13.1	39.8 ± 14.3	40.0 ± 15.9	41.2 ± 13.2	40.1 ± 9.9
**LA-V_preA_ (mL)**	26.5 ± 11.4	27.8 ± 12.3	28.9 ± 9.3	30.2 ± 10.1	28.2 ± 11.7	23.8 ± 13.2	31.3 ± 14.5	27.8 ± 11.6	23.8 ± 8.8 ‡
**LA-V_min_ (mL)**	20.6 ± 8.7	19.3 ± 8.4	18.8 ± 5.8	24.3 ± 8.5	19.7 ± 8.0 †	13.4 ± 4.9 †/††	24.0 ± 11.6	19.4 ± 7.3 ‡	15.2 ± 5.2 ‡/‡‡
**basal LA-RS (%)**	−10.4 ± 5.9	−16.8 ± 6.7 ∗	−33.8 ± 8.5 ∗/∗∗	−14.2 ± 8.3	−18.6 ± 9.4 †	−17.7 ± 8.2	−15.7 ± 7.9	−18.5 ± 9.7	−16.7 ± 7.1
**LA-GRS (%)**	−3.1 ± 2.1	−14.9 ± 4.7 ∗	−29.6 ± 6.6 ∗/∗∗	−10.0 ± 6.8	−15.0 ± 8.1 †	−18.2 ± 7.5 †/††	−10.2 ± 9.0	−15.4 ± 7.9 ‡	−16.0 ± 6.0 ‡
**basal LA-CS (%)**	32.1 ± 11.4	42.5 ± 14.7 ∗	50.4 ± 17.8 ∗/∗∗	24.9 ± 6.2	40.9 ± 10.2 †	60.9 ± 17.8 †/††	32.5 ± 13.5	42.6 ± 14.8 ‡	48.4 ± 15.9 ‡
**LA-GCS (%)**	24.1 ± 10.4	34.6± 15.2 ∗	38.3 ± 17.2 ∗/∗∗	13.4 ± 3.2	31.7 ± 7.7 †	58.6 ± 11.0 †/††	22.1 ± 12.1	33.7 ± 14.1 ‡	43.0 ± 15.9 ‡
**basal LA-LS (%)**	18.2 ± 8.6	24.1 ± 11.4 ∗	19.0 ± 7.9	18.7 ± 11.8	22.6 ± 10.3	26.0 ± 11.8 †	12.4 ± 5.3	22.6 ± 9.9 ‡	32.6 ± 10.5 ‡/‡‡
**LA-GLS (%)**	19.5 ± 8.3	27.6 ± 8.6 ∗	25.3 ± 7.6	17.9± 5.3	26.6 ± 8.0 †	31.7 ± 9.5 †/††	13.3 ± 3.3	25.6 ± 4.6 ‡	40.4 ± 5.9 ‡/‡‡
**LV-EDV (mL)**	82.2 ± 25.1	86.1± 22.9	91.4 ± 3.9	76.8 ± 28.6	87.3 ± 20.7 †	91.5 ± 22.3 †	86.0 ± 4.9	85.8 ± 23.6	88.4 ± 16.2
**LV-ESV (mL)**	34.3 ± 12.1	36.3 ± 9.7	38.9 ± 12.1	32.1 ± 11.3	37.3 ± 9.4 †	36.7±12.1	37.7 ± 11.7	35.9 ± 10.6	37.2 ± 8.0
**LV-EF (%)**	58.8± 5.5	58.0 ± 5.2	57.7 ± 8.3	60.2 ± 5.6	57.2 ± 5.1 †	60.0 ± 7.3 ††	56.8 ± 5.9	58.4 ± 5.7	57.9 ± 4.7
**LV mass (g)**	154.7 ± 31.1	157.6± 32.4	175.6 ± 25.1 ∗∗	156.8 ± 25.2	160.2 ± 32.3	158.1±36.2	165.4 ± 30.4	160.0 ± 31.5	150.7 ± 34.6
**basal LV-RS (%)**	32.6 ± 12.8	31.1 ± 12.3	35.4 ± 16.8	33.5 ± 12.3	30.3± 12.7	36.6± 13.5 ††	32.7 ± 14.5	32.2 ± 12.4	28.5 ± 13.7
**LV-GRS (%)**	24.9 ± 9.6	25.1 ± 8.6	28.9± 13.8	27.0± 9.6	24.6 ± 8.7	28.1± 11.7	24.7 ± 10.7	25.7 ± 9.4	24.8 ± 8.8
**basal LV-CS (%)**	−26.7 ± 4.4	−25.2 ± 5.3	−26.0 ± 5.9	−27.1 ± 4.6	−25.1 ± 4.6†	−26.0 ± 7.3	−25.5 ± 5.1	−25.7 ± 5.3	−24.3 ± 5.1
**LV-GCS (%)**	−28.1 ± 5.2	−27.5 ± 4.8	−27.1 ± 6.7	−29.3 ±4.8	−26.9± 4.7†	−28.5 ± 6.3	−26.6 ± 5.4	−27.7 ± 5.2	−27.5 ± 4.1
**basal LV-LS (%)**	−20.0 ± 4.1	−19.7 ± 4.5	−21.6 ± 5.1	−20.7 ± 4.5	−19.8 ± 4.4	−20.0 ± 5.2	−20.4 ± 4.7	−20.0 ± 4.6	−19.1 ± 3.8
**LV-GLS (%)**	−16.5 ± 3.0	−16.2 ± 2.3	−15.5 ± 2.4	−16.9 ± 2.6	−16.0 ± 2.3	−16.2 ± 2.8	−15.5 ± 2.5	−16.4 ± 2.4	−16.0 ± 2.4

* *p* < 0.05 vs. LA-GRS < −6.6%. ** *p* < 0.05 vs. −6.6% ≤ LA-GRS ≤ −23%. † *p* < 0.05 vs. LA-GCS < 18.2%. †† *p* < 0.05 vs. 18.2% ≤ LA-GCS ≤ 48.8%. ‡ *p* < 0.05 vs. LA-GLS < 17.2%. ‡‡ *p* < 0.05 vs. 17.2% ≤ LA-GLS ≤ 35%. **Abbreviations.** LA = left atrial; LV = left ventricular; Vmax = maximum LA volume; VpreA = pre-atrial contraction LA volume; Vmin = minimum LA volume; RS = radial strain; CS = circumferential strain; LS = longitudinal strain; GRS = global radial strain; GCS = global circumferential strain; GLS = global longitudinal strain; EDV = end-diastolic volume; ESV = end-systolic volume; EF = ejection fraction.

**Table 3:** Left atrial and left ventricular volumes and end-systolic strains in different left ventricular strain groups.

	LV-GRS < 16.1% (*n* = 25)	16.1% ≤ LV-GRS ≤ 34.9% (*n* = 115)	34.9% < LV-GRS (*n* = 25)	LV-GCS < −22.4% (*n* = 16)	−22.4% ≤ LV-GCS ≤ −32.6% (*n* = 122)	−32.6% < LV-GCS (*n* = 27)	LV-GLS < −13.8% (*n* = 25)	−13.8% ≤ LV-GLS ≤ −18.6% (*n* = 116)	−18.6% < LV-GLS (*n* = 24)
**LA-V_max_ (mL)**	39.6 ± 9.9	41.8 ± 14.0	37.7 ± 11.6	45.0 ± 11.3	41.00 ± 13.5	38.1 ± 12.3†	43.1 ± 14.1	40.8 ± 13.2	39.7 ± 12.3
**LA-V_preA_ (mL)**	25.8 ± 7.9	28.7 ± 12.9	25.1 ± 9.7	30.3 ± 12.2	28.0 ± 12.3	25.0 ± 9.6	30.6 ± 14.0	27.4 ± 12.0	27.0 ± 8.9
**LA-V_min_ (mL)**	18.4 ± 5.7	19.9 ± 8.6	17.9 ± 8.5	21.0 ± 8.2	19.5 ± 8.6	17.6 ± 6.4	21.3 ± 10.8	19.1 ± 7.8	19.5 ± 6.9
**basal LA-RS (%)**	−17.2 ± 9.2	−17.4 ± 8.6	−19.9 ± 11.3	−23.0 ± 8.8	−17.2 ± 8.9	−17.6 ± 9.3†	−17.1 ± 8.4	−18.3 ± 9.2	−16.2 ± 9.3
**LA-GRS (%)**	−12.9 ± 8.4	−14.7 ± 7.6	−17.4 ± 10.0 *	−19.6 ± 10.0	−14.3 ± 7.5 †	−14.8 ± 8.9	−15.3 ± 8.3	−14.9 ± 8.0	−14.0 ± 8.6
**basal LA-CS (%)**	42.4 ± 12.4	41.7 ± 16.1	42.2 ± 14.8	45.5 ± 16.8	41.3 ± 14.5	44.6 ± 20.1	40.3 ± 14.8	42.8 ± 14.0	38.8 ± 20.8
**LA-GCS (%)**	34.2 ± 13.4	33.2 ± 15.6	34.2 ± 16.8	37.9 ± 17.0	32.6 ± 14.1	37.1 ± 20.2	35.1 ± 14.9	33.5 ± 14.3	32.2 ± 20.4
**basal LA-LS (%)**	24.2 ± 10.8	22.9 ± 11.1	20.4 ± 10.6	17.7 ± 10.5	23.4 ± 10.5 †	20.7 ± 10.5	16.7 ± 7.6	23.2 ± 10.5 ‡	26.1 ± 13.6 ‡
**LA-GLS (%)**	25.2 ± 7.2	26.2 ± 8.6	27.0 ± 11.6	24.0 ± 9.3	26.4 ± 9.1	25.9 ± 7.9	22.9 ± 7.7	26.7 ± 8.5 ‡	26.6 ± 11.1
**LV-EDV (mL)**	81.5 ± 19.2	86.7 ± 24.2	88.6 ± 19.9	82.6 ± 15.7	87.5 ± 24.2	82.6 ± 19.7	91.4 ± 25.5	85.9 ± 21.7	79.9 ± 24.2
**LV-ESV (mL)**	36.1 ± 6.8	36.8 ± 10.1	34.3 ± 11.1	39.7 ± 8.2	37.8 ± 10.0	27.7 ± 8.7 ††	40.2 ± 12.9	36.3 ± 9.5	31.9 ± 9.5 ‡/‡‡
**LV-EF (%)**	55.1 ± 4.9	57.9 ± 5.2 *	61.9 ± 6.5 */**	51.8 ± 4.7	57.0 ± 3.4 †	66.9 ± 4.7 †/††	56.1 ± 5.3	57.7 ± 5.3	62.0 ± 6.3 ‡/‡‡
**LV mass (g)**	155 ± 27.6	159.3 ± 32.8	162.0 ± 33.1	162.8 ± 30.8	160.3 ± 31.9	150.5 ± 33.0	172.3 ± 33.5	157.6 ± 32.0 ‡	152.8± 25.0 ‡
**basal LV-RS (%)**	19.6 ± 8.2	30.4 ± 9.4 *	50.3 ± 11.9 */**	29.3 ± 11.8	30.6 ± 12.4	38.0 ± 14.5 †/††	35.1 ± 15.3	30.6 ± 11.8	33.9 ± 14.3
**LV-GRS (%)**	12.7 ± 3.3	24.6 ± 4.7 *	42.0 ± 6.6 */**	19.6 ± 7.3	24.5 ± 8.3†	33.4 ± 11.0 †/††	26.7 ± 11.6	24.5 ± 8.6	28.4 ± 10.3 ‡‡
**basal LV-CS (%)**	−23.9 ± 4.4	−25.0 ± 5.2	−29.2 ± 4.9 */**	−21.0 ± 4.8	−24.7 ± 4.4†	−31.3 ± 4.6 †/††	−26.6 ± 5.0	−25.1 ± 5.0	−26.4 ± 6.1
**LV-GCS (%)**	−25.1 ± 4.5	−27.4 ± 4.7 *	−30.7 ± 6.0 */**	−19.4 ± 2.2	−26.7 ± 2.8†	−36.0 ± 2.8 †/††	−26.2 ± 5.0	−27.1 ± 4.8	−30.3 ± 6.0 ‡/‡‡
**basal LV-LS (%)**	−19.3 ± 4.0	−19.8 ± 4.6	−21.0 ± 4.5	−20.3 ± 5.5	−19.9 ± 4.4	−19.8 ± 5.1	−17.5 ± 4.0	−19.9 ± 4.1‡	−23.1 ± 5.2 ‡/‡‡
**LV-GLS (%)**	−15.5 ± 2.1	−16.3 ± 2.3	−16.2 ± 30.2	−15.6 ± 2.2	−16.0 ± 2.2	−17.0 ± 3.3	−12.7 ± 1.4	−16.2 ± 1.4‡	−20.0 ± 1.1 ‡/‡‡

* *p* < 0.05 vs. LV-GRS < 16.1%. ** *p* < 0.05 vs. 16.1% ≤ LV-GRS ≤ 34.9%. † *p* < 0.05 vs. LV-GCS < −21.9%. †† *p* < 0.05 vs. −21.9% ≤ LV-GCS ≤ −32.6%. ‡ *p* < 0.05 vs. LV-GLS < −13.8%. ‡‡ *p* < 0.05 vs. −13.8% ≤ LV-GLS ≤ −18.6%. **Abbreviations.** LA = left atrial; LV = left ventricular; Vmax = maximum LA volume; VpreA = pre-atrial contraction LA volume; Vmin = minimum LA volume; RS = radial strain; CS = circumferential strain; LS = longitudinal strain; GRS = global radial strain; GCS = global circumferential strain; GLS = global longitudinal strain; EDV = end-diastolic volume; ESV = end-systolic volume; EF = ejection fraction.

## Data Availability

The original contributions presented in the study are included in the article, further inquiries can be directed to the corresponding author.
